# Treatment of Hepatitis C Post-Liver Transplantation Could Mitigate Discard Rates of Hepatitis C-Positive Deceased Donor Livers and Expand the Donor Pool

**DOI:** 10.1155/2021/6612453

**Published:** 2021-01-25

**Authors:** Jennifer Keller, Gary Marklin, Obi Okoye, Roshani Desai, Tej Sura, Ajay Jain, Chintalapati Varma, Mustafa Nazzal

**Affiliations:** ^1^Division of Abdominal Transplant, Saint Louis University, St. Louis, MO, USA; ^2^Mid-America Transplant Services, St. Louis, MO, USA; ^3^Division of Hepatology, Saint Louis University, St. Louis, MO, USA; ^4^Saint Louis University School of Medicine, St. Louis, MO, USA

## Abstract

**Background:**

Prior to 2014, treatment for hepatitis C was limited. However, the subsequent introduction of direct acting antiviral medications (DAA) against hepatitis C led to improvements in morbidity and better medication tolerance. DAA therapy allowed for an increase in treatment rates of hepatitis C in patients on the liver transplant waiting list. With the popularization of DAA, there became a growing concern about the utility of hepatitis C-positive (HCV+) deceased liver donors, especially after treating HCV+ potential recipients on the transplant waiting list.

**Methods:**

This is a retrospective, observational study using Mid-America Transplant Services (MTS) database from 2008 to 2017. Comparison was made before the widespread use of DAAs 2008–2013 (pre-DAA) against their common practice use 2014–2017 (post-DAA). All deceased liver donors with HCV antibody or nucleic acid positive results were evaluated.

**Results:**

Between 2008 and 2017, 96 deceased liver donors were positive for HCV. In the pre-DAA era, 47 deceased liver donors were positive for HCV, of which 32 (68.1%) were transplanted and 15 (31.9%) were discarded. In the post-DAA era, a total of 49 HCV+ organs were identified, out of which 43 (87.8%) livers were transplanted and 6 (12.2%) were discarded. Discard rate was significantly higher in the pre-DAA population (31.9% vs. 12.2%, *p* = 0.026). Secondary analysis showed a distinct trend towards increased regional sharing and utilization of HCV+ donors.

**Conclusion:**

In order to reduce discard rates of HCV+ patients, our data suggest that transplant centers could potentially delay HCV treatment in patients on the transplant waitlist.

## 1. Introduction

Hepatitis C virus (HCV) was a leading cause of liver cirrhosis and one of the most common indications for liver transplantation (LT). Unfortunately, LT is not a treatment for HCV in the recipient, and the recurrence of HCV is almost certain [1, 2]. Following liver transplantation, approximately 5% of patients with HCV develop fibrosing cholestatic hepatitis, an aggressive and devastating complication. Fibrosing cholestatic hepatitis can lead to rapid organ loss associated with high mortality [3]. Though these risks exist, multiple trials have shown that the use of HCV+ donors in HCV+ recipients carries no significant differences in graft survival or mortality rates when compared to non-HCV donors [4]. Although some transplant centers have carefully utilized HVC-positive deceased liver donors—in the absence of advanced fibrosis or significant steatosis—to transplant HCV+ recipients, such a practice has not been uniformly adopted across all centers. Indeed, the decision of when to treat HCV in patients, pre or posttransplant, is still a center specific decision.

Prior to 2013, the treatment for HCV consisted of interferon/peginterferon and ribavirin which yielded a sustained virologic response (SVR) of 55%, depending on genotype, patient characteristics, and viral load [3, 5]. This poor response to treatment, along with the side effects, was responsible for the reluctance of transplant centers to utilize HCV+ deceased donors. Another concern of using HCV+ deceased donors was the risk of transmitting a more resistant HCV genotype [6, 7]. The inability to perform genotype testing on the donor prior to transplantation exacerbated these concerns. Given these predicaments, it was the policy of some centers to only transplant recipients with genotype 1 or 4 with HCV+ donors. Policies like these prevented transmission of a more resistant genotype to a recipient with HCV genotypes 2 or 3, which were easier to treat in the interferon era.

The introduction of direct acting antiviral (DAA) medications has revolutionized the treatment of HCV. In comparison to interferon and ribavirin, DAAs have improved rates of sustained virologic response including in those with historically adverse genotypes [8]. The higher rates of SVR have led to decreased morbidity and possibly mortality associated with HCV [9]. DAAs have been used in the treatment of HCV in liver transplant recipients. Importantly, such treatment was not associated with death or graft failure. Moreover, DAAs are increasingly being used to treat HCV+ patients while on the liver transplant waiting list, thus reducing the number of potential recipients on the transplant list with HCV [10].

With the availability of an effective treatment for HCV+ recipients on the transplant list and subsequent reduction of HCV+ waitlist members, there was in parallel a concern for the decline of HCV+ donors. In theory, this would lead to the reduction in the transplantation of HCV+ donors to HCV+ recipients, with a resultant increase in the discard rate. In order to objectively quantify this metric, we conducted a retrospective study in our local Organ Procurement Organization (OPO) to evaluate the discard rate of HCV+ deceased liver donors in the era prior to DAA and compared it to the time following the introduction of DAAs.

## 2. Methods

This was a retrospective, observational study using Mid-America Transplant Services (MTS) database, our local OPO. Deceased donors' identifiers including sex were removed. The study took place between 2008 and 2017. Utilizing this study period allowed us to compare a time before the widespread use of DAAs (2008–2013) and a time where DAAs were more common practice (2014–2017). All deceased donors with HCV antibody or nucleic acid positive results were evaluated.

Statistical analysis: Graph Pad Prism version 7.03 software was utilized for statistical analysis. Descriptive statistics on the outcomes were calculated as mean, median, and interquartile range (IQR). A chi-square analysis was employed to compare the two outcome eras. Statistical significance was considered upon a *p* value of less than 0.05. A subgroup analysis was run using binary logistic regression to evaluate for differences between donor factors.

## 3. Results

### 3.1. Donor Characteristics

Between 2008 and 2017, 96 organ donors tested positive for HCV with a median age of 38.5 years (1^st^ quartile, 28 and 3^rd^ quartile, 48.25). Among the causes of death, drug intoxication was the most common (34.38%) followed by intracranial hemorrhage/stroke (23.96%). Cardiovascular disease accounted for 8.33% donors, and this group also had the highest median age of 49 years (Table 1).

### 3.2. Discard Rate Comparisons

The discard rate was compared between 2008 through 2013 (pre-DAA) and 2014 through 2017 (post-DAA). In the pre-DAA era, a total of 47 organ donors tested positive for HCV, of which 32 (68.1%) were transplanted and 15 (31.9%) were discarded. In the post-DAA era, a total of 49 HCV+ organs were identified. Of the 49, 43 (87.8%) of the livers were transplanted and 6 (12.2%) were discarded (Table 2). We noted that the discard rate was significantly higher in the pre-DAA population (*p* = 0.026).

### 3.3. Donor Risk Adjustment

In order to attribute liver discards to hepatitis C status, the discard rates were adjusted for donor risk factors. The adjusted risk factors included in the analysis were age, diabetes, and hypertension (Table 3). We noted that other than age (*p* = 0.025), no other donor risk factors reached statistical significance.

### 3.4. Organ Distribution

A secondary subgroup analysis was performed to examine the location where organs were allocated and categorized into locally or shared regionally. Once again, the two different eras were compared. In pre-DAA years, 19 livers were transplanted locally, whereas 13 livers were shared regionally. In the post-DAA years, 17 livers were transplanted locally, and 26 livers were shared for regional transplantation. Overall, 36 livers were transplanted locally. Out of these, 59.4% of them were transplanted in the pre-DAA era, and 39.5% of them were transplanted in the post-DAA era. In comparison, overall 39 livers were shared for transplantation over the region with 40.6% of them being utilized in the pre-DAA era and 60.5% of were transplanted in the post-DAA era. Though not statistically significant, there was a trend to increased regional sharing of hepatitis C donor organs (40% vs. 60.5%, *p* = 0.106) (Table 4).

## 4. Discussion

Advances in organ utilization and sharing have improved the availability of liver transplantation, but roughly 11,000 candidates still remain on the waiting list, while only 8000 organs are available for transplantation [11]. The shortage of donors necessitates the utilization of different strategies to increase the donor pool and to reduce organ discard rates [12]. In 2017, our OPO had 12.2% HCV-positive donors, with 9.4% testing positive with nucleic acid testing (NAT) and antibody (Ab) positive. 2.8% was Ab-positive and NAT-negative donors. Most of these donors were younger than 40 years of age. This high number of HCV+ donors may be related to the lack of access to medical care, high expense of DAAs, and the heroin epidemic [13]. The high rate of HCV+ donors nevertheless could be a valuable donor pool. The increased treatment of HCV in the pretransplant setting created a growing concern that the HCV+ organ discard rate would increase due to fewer HCV+ recipients.

Treatment of pretransplant, waitlisted cirrhotic patients appears to be efficacious and attractive in the new era of DAA. The high SVR rate that has been shown with DAA can potentially mean improved liver function, prevention of decompensation, and posttransplant HCV recurrence [14]. However, there are also disadvantages to treating HCV while on the waitlist. SVR may only lead to a minute improvement in the model of end stage liver disease score (MELD) without significant improvement of the sequelae of portal hypertension, including ascites and encephalopathy. This leads to the so-called ”MELD Purgatory,” where the improvement in liver function is not enough to get the patient off the list, yet the patient has lost priority on the waiting list [14, 15]. The pretransplantation treatment also begs the logical concern of decreasing the number of HCV+ patients on the transplant list, potentially leading to a discard of the HCV+ donors. Given these factors, some centers are utilizing HCV+ donors for HCV− recipients in order to transplant patients with a lower MELD, reduce discard rate, and reduce patient wait time [16]. Most studies assessing transplantation of HCV+ donors into HCV− recipients have been performed in renal transplantation. Recently, the use of HCV-seropositive, nonviremic liver donors into seronegative recipients is being carried out in some transplant centers [17–20].

Our study showed that in our local OPO, it appeared that the discard rate was higher in the pre-DAA era in comparison to the post-DAA era. A secondary analysis showed that the number of HCV+ organs that were transplanted regionally was much higher than those transplanted locally in the post-DAA era in comparison to the pre-DAA era. The decrease in discard rate of HVC+ organs in our OPO is related to the introduction of highly efficacious DAAs. Over the last four years, the concern of using these organs has decreased. This study also noted a shift of organ utilization with an increase in the regional use of HCV-positive donors comparted to the local centers. This is mostly related to center-derived variation in utilizing HCV-positive organs differently and is likely a reflection of waitlist HCV management. Centers that do not treat HCV+ potential recipients on the liver transplant waitlist will have a higher number of patients that will be able to receive HCV+ donors.

A limitation of this study was unavailability of objective data about the recipient outcome using HCV-positive donors or on the numbers of HCV RNA-positive donors used for HCV RNA-negative recipients. However, as has been highlighted, even early on, with the introduction of DAA and before any concrete data in transplant recipients was widely available, transplant physicians were using higher numbers of organs from DAA-treated HCV patient and not necessarily using it in those who were transplanted.

## 5. Conclusions

DAAs has been an extremely effective modality in the treatment of HCV with minimal side effects.

Indeed, HCV+ donors compose a high percentage of the current deceased donor pool. Most of these donors are young donors with a greater chance of high-quality organs. In light of the current waitlist, it is critical to utilize all these viable organs in an effort to reduce shortage of organs. By abstaining from the treatment of HCV+ waitlist patients, we might give patients the opportunity to be transplanted at a lower MELD score and decrease the discard rate of HCV+ organs. In the current era, we thus recommend the use of DAAs for the treatment of hepatitis C in the posttransplant setting to better utilize available organs.

## Figures and Tables

**Table 1:** Patient characteristics.

Mechanism of death	Median age	Number	Percentage (%)
Asphyxiation	34	4	4.17
Blunt injury	42	13	13.54
Cardiovascular	49	8	8.33
Drug/intoxication	29	33	34.38
Gunshot wound	35	12	12.50
Intracranial hemorrhage/stroke	46	23	23.96
Others	45	3	3.13

**Table 2:** Distribution of organs over time in study population.

	2008–2013 (*n* = 47)	2014–2017 (*n* = 49)	*p*
Transplanted	32 (68.1%)	43 (87.8%)	**0.026**
Discarded	15 (31.9%)	6 (12.2%)	

Bold signifies a statistically significant P value.

**Table 3:** Donor risk factors of transplanted organ distribution.

Factor	Odds ratio	*p*
Age	0.95 (0.90, 0.99)	**0.025**
Hypertension	1.25 (0.22, 7.17)	0.803
Alcohol	3.61 (0.66, 19.82)	0.139
Pre-DAA era	1.40 (0.29, 6.77)	0.677

Bold signifies a statistically significant P value.

**Table 4:** Distribution of transplanted organs.

	Local (*n* = 36)	Regional (*n* = 39)	*p*
2008–2013	19 (59.4%)	13 (40.6%)	0.106
2014–2017	17 (39.5%)	26 (60.5%)	

## Data Availability

The data used to support the findings of this study are included within the article.
